# Nanopesticide Formulation from Pyraclostrobin and Graphene Oxide as a Nanocarrier and Application in Controlling Plant Fungal Pathogens

**DOI:** 10.3390/nano12071112

**Published:** 2022-03-28

**Authors:** Fei Peng, Xiuping Wang, Wenjing Zhang, Xuejuan Shi, Caihong Cheng, Wenlong Hou, Xiaohu Lin, Xiaolu Xiao, Jun Li

**Affiliations:** 1 Hebei Key Laboratory of Active Components and Functions in Natural Products, Hebei Normal University of Science and Technology, Qinhuangdao 066004, China; flyer5212528@163.com (F.P.); zwj19990123@126.com (W.Z.); cch20059@126.com (C.C.); wenlonghou@126.com (W.H.); 2 Analysis and Testing Center, Hebei Normal University of Science and Technology, Qinhuangdao 066000, China; wangxiuping0721@163.com (X.W.); xiaohulin2008@163.com (X.L.); 3 College of Agronomy and Biotechnology, Hebei Normal University of Science and Technology, Qinhuangdao 066000, China; xuejuanshi@163.com; 4 Key Laboratory of Biology and Genetic Improvement of Oil Crops of Ministry of Agriculture and Rural Affairs, Oil Crops Research Institute of the Chinese Academy of Agricultural Sciences, Wuhan 430062, China; xiaoxiaoluxt@163.com

**Keywords:** graphene oxide, nanocarrier, nanopesticide, pathogenic fungal control

## Abstract

Efficient and environment-friendly nanopesticide delivery systems are critical for the sustainable development of agriculture. In this study, a graphene oxide nanocomposite was developed for pesticide delivery and plant protection with pyraclostrobin as the model pesticide. First, graphene oxide–pyraclostrobin nanocomposite was prepared through fast adsorption of pyraclostrobin onto graphene oxide with a maximum loading of 87.04%. The as-prepared graphene oxide–pyraclostrobin nanocomposite exhibited high stability during two years of storage, suggesting its high potential in practical application. The graphene oxide–pyraclostrobin nanocomposite could achieve temperature (25 °C, 30 °C and 35 °C) and pH (5, 7 and 9) slow-release behavior, which overcomes the burst release of conventional pyraclostrobin formulation. Furthermore, graphene oxide–pyraclostrobin nanocomposite exhibited considerable antifungal activities against *Fusarium graminearum* and *Sclerotinia sclerotiorum* both in vitro and in vivo. The cotoxicity factor assay revealed that there was a synergistic interaction when graphene oxide and pyraclostrobin were combined at the ratio of 1:1 against the mycelial growth of *Fusarium graminearum* and *Sclerotinia sclerotiorum* with co-toxicity coefficient values exceeding 100 in vitro. The control efficacy of graphene oxide–pyraclostrobin nanocomposite was 71.35% and 62.32% against *Fusarium graminearum* and *Sclerotinia sclerotiorum* in greenhouse, respectively, which was higher than that of single graphene oxide and pyraclostrobin. In general, the present study provides a candidate nanoformulation for pathogenic fungal control in plants, and may also expand the application of graphene oxide materials in controlling plant fungal pathogens and sustainable agriculture.

## 1. Introduction

Pesticides play important roles in controlling plant diseases, weeds and insects to ensure crop productivity and promote the sustainable development of agriculture [1,2,3]. It has been reported that nearly 3.5 million tons of synthetic pesticides are applied every year to control pests worldwide [4,5]. In fact, only less than 0.1% of pesticides can reach the targets, while more than 99% of them cannot exert their bioactivity since most of them are lost due to leaching, evaporation and drifting or degradation by light, heat and microorganisms [6,7,8,9]. In addition, conventional pesticide formulation involves the use of large amounts of organic solvents, posing serious threats of pollution to the environment and toxicity to non-target organisms [10,11,12]. Therefore, it is urgent to develop appropriate pesticide delivery systems and high-efficiency and green pesticide formulations, which may help to reduce the dosage of pesticides and improve their efficacy.

In recent years, smart nano-delivery systems of pesticides have attracted increasing attention [13,14]. By more scientific and rational design, the pesticide delivery system involving on-demand or site-specific release with sustained bioactivity could minimize or avoid the repeated application of pesticides [15,16]. Besides, it can also decrease the environmental risk of pesticides by reducing the application dosage and frequency of pesticides. Various nanomaterials such as graphene-based nanomaterials, mesoporous silica nanoparticles and metal-inorganic materials have been developed as vectors for pesticide delivery [17,18,19]. Among the various nanocarriers developed for pesticides, graphene oxide (GO) is considered as a robust scaffold for pesticide delivery owing to its advantages including easy surface modification, high loading rate, large surface area and good water solubility [20,21]. For example, Song et al. reported the application of GO as a carrier to deliver emamect in benzoate, which can enhance the dispersion stability of pesticides and the sustainable antipest activity [22]. Tong et al. demonstrated that polydopamine-coated GO has higher loading capacity for hymexazol with pH-controlled release [23]. It has been proved that GO can enhance the leaf affinity of pesticides as a nanocarrier to reduce the loss of applied pesticides [23,24,25]. However, in real application, the pesticide formulated with nanocarriers should have simple components, easy preparation process, multifunctionality and high bioactivity, as well as appropriate pesticide content, storage stability and sustained effects.

Pyraclostrobin (Pyr), a broad-spectrum, high-efficiency and low-toxicity novel strobilurin fungicide, has protective and curative effects on crops [26]. Herein, we combined the existing Pyr pesticide with GO to formulate new GO–Pyr nanopesticide, and tested its antifungal activity against two important plant diseases wheat scab and rape sclerotinia infected by pathogenic fungi *Fusarium graminearum* (FG) and *Sclerotinia sclerotiorum* (SS). Pyr was loaded on a GO nanocarrier through a physisorption process, forming a GO–Pyr nanocomposite with high antifungal activity. In consideration of the high efficiency, simple preparation, multifunction and high biosafety, GO holds great promise in smart pesticide delivery and plant protection.

## 2. Materials and Methods

### 2.1. Materials

Graphite was provided by Qingdao Tianhe Graphite Co. Ltd. (Qingdao, China). The average particle diameter was 4 mm (99.95% of purity). All other reagents were of HPLC grade and purchased from the Tianjin No. 3 Chemical Plant. Pyr (ACS grade) was provided by Sigma-Aldrich (Shanghai, China).

### 2.2. Characterization

Transmission electron microscopy (TEM) of GO was performed by Tecnai G20 microscopy (FEI, Czech, Tokyo, Japan). The size and morphology of GO were examined by atomic force microscope (AFM, Nanoscope IIIa, Veeco Instruments Inc., Plainview, NY, USA). The specific surface area of the GO was determined by a surface area analyzer (JW-K, Beijing, China) through the Brunauer–Emmett–Teller (BET) method with an adsorbent of N_2_. Scanning electron microscopy (SEM, Hitachi, SU8010, Tokyo, Japan) was conducted to investigate the morphology of GO and GO–Pyr nanocomposites, and the infrared absorption spectra were collected on a Fourier transform infrared (FT-IR) spectroscope (Bruker, TENSOR-27, Karlsruhe, Germany). Thermogravimetric analysis (TGA) was carried out with a STA 409 PC (Netzsch, Bavaria, Germany) from 25 °C to 700 °C at a heating rate of 10 °C min^−1^ under N^2^ atmosphere. GO–Pyr was placed on paraffin film, and the contact angle (Dataphysics, OCA20, Stuttgart, Germany) was measured.

### 2.3. Fungal Strains

Plant pathogenic fungus FG was obtained from the College of Plant Science and Technology of Huazhong Agricultural University. SS was obtained from Oil Crops Research Institute of the Chinese Academy of Agricultural Sciences. The fungal cultures were routinely maintained on a potato dextrose agar (PDA, Nantong Kaiheng Biotechnology Development Co., Ltd, Nantong, Jiangsu Province, China) slant at 4 °C. 

### 2.4. Preparation of Graphene Oxide–Pyraclostrobin Nanocomposites

First, GO was prepared with the Hummer’s method [27]. Physical loading of Pyr onto the surface of GO was conducted to obtain the GO–Pyr nanocomposite [28]. For screening the optimal combination ratio between GO and Pyr, the inhibitory effects of GO–Pyr combined at different ratios (1:9, 2:8, 3:7, 6:4, 5:5, 4:6, 7:3, 8:2 and 9:1) on the mycelial growth of FG was tested. As a result, the ratio of 5:5 resulted in the best bioactivity (Figure S1). Therefore, GO–Pyr combined at 5:5 was selected as the optimal combination for subsequent preparation, characterization and bioactivity analysis. To be specific, 500 μg Pyr was dispersed in a mixture (2 mL) of methanol, Tween 20 and water (MT, 1: 1: 98, *v*/*v*). Then, 500 μg GO was added into the mixture to obtain a final GO/Pyr ratio of 1:1, which was then stirred in the dark for 24 h. The generated products were washed with deionized water and then freeze-dried for further use. 

### 2.5. Determination of the Pesticide Loading Capacity of Graphene Oxide

The loading content (LC) of Pyr on the surface of GO was measured using HPLC (Thermo Fisher LTQ Orbitrap XL, Waltham, MS, USA). In brief, the GO–Pyr nanocomposite was dissolved in 25.0 mL of methanol with vigorous vortexing, and the clear solution was collected to perform HPLC analysis. The HPLC parameters were: ZORBAX Eclipse Plus C_18_ column (250 mm × 4.6 mm, 5 μm; Agilent, Palo Alto, CA, USA) with UV detection at 275 nm. A flow rate of 1 mL/min was used with a mobile phase composed of methanol and water (80:20, *v*/*v*), and the injection volume was 10 μL. The pesticide LC was obtained with the following equation [29]:

*LC* (%) = *W_pyr_*/*W_GO_* × 100 (1)

where *W_pyr_* is the weight of Pyr loaded on GO (μg), and *W_GO_* is the weight of GO (μg).

### 2.6. In Vitro Release Experiment

The release behavior of Pyr was studied as follows. About 5 mL dispersion of GO–Pyr nanocomposite was placed in a dialysis bag (molecular weight cutoff = 3500 Da) (Mym Biological Technology Co., Ltd, Chicago, IL, USA), which was subsequently placed in 45 mL methanol–water mixture (1:1, *v*/*v*) in a centrifuge tube. The centrifuge tube was shaken at 200 rpm and the temperature of 25 °C, 30 °C and 35 °C. At the predetermined time points, 1 mL of supernatant was collected, with the addition of 1 mL fresh medium each time. The solution was filtered with a cellulose-membrane filter (diameter, 13 mm; pore size, 0.22 μm; Dikma China Limit Technologies Inc., BeiJing, China) and then injected into the HPLC system to measure the pesticide concentration. The Korsmeyer–Peppas model Equation (2) and Higuchi model Equation (3) were employed for analysis of the Pyr release behavior from GO:

*M_t_*/*M*_∞_ = *kt*^n^ (2)

*M_t_*/*M*_∞_ = *kt*^0.5^ (3)

where *M_t_* (mg) is the cumulative release at a certain time points, *M_∞_* (mg) represents the total Pyr release amount at equilibrium, *k* (d^−n^) indicates the kinetic constant, *t* (d) is the time point, and n stands for a constant associated with the release mechanism [30].

To investigate the pH-dependent release performance of Pyr, the pH of 30% ethanol aqueous solution was adjusted to 5.0, 7.0 and 9.0 by PBS buffer. Other operations were the same as the above process. 

### 2.7. Stability Test

#### 2.7.1. Storage Stability at Low or High Temperature

The solution of GO–Pyr nanocomposite (containing 1 mg/mL Pyr) was put in brown glass bottles and maintained at 0 °C for 7 d or at 54 °C for 14 d. Then, the Pyr content was determined with HPLC [31].

#### 2.7.2. Long-Term Storage Stability

The solution of GO–Pyr nanocomposite was put into glass bottles and placed in dark, dry and ventilated places for 24 months. The Pyr content was determined every four months by HPLC [32].

### 2.8. Bioassay of the Antifungal Activity of Graphene Oxide–Pyraclostrobin Nanocomposite In Vitro

To test the in vitro antifungal effect, the antifungal activity of Pyr alone or combined with GO on the mycelial growth of FG and SS was determined. In brief, FG or SS was loaded onto solid PDA containing GO, Pyr or GO–Pyr at different concentrations. MT solution at an equal volume without any fungicide was also processed to serve as the control. After incubation at 24 ± 2 °C for 120 h, the mycelial growth of FG and SS was observed. The rate of mycelial growth inhibition (*I*, %) was calculated by using the following equation:

*I* = (1 − *Dt*/*Dc*) × 100% (4)

In the equation, *Dc* and *Dt* are the mycelial diameter or biomass of the control and treatment after 120 h of incubation, respectively [33]. The antifungal activity was measured through a totally random design with four replications.

### 2.9. Control Efficacy of Graphene Oxide–pyraclostrobin Nanocomposite In Vivo

Greenhouse bioassays were performed to determine the antifungal activity of Pyr alone or combined with GO. The seeds of edible rape (*Brassica rapa* L.) Zhong Huaqing were purchased from the College of Agriculture and Biotechnology, Zhejiang University. The seeds of wheat cultivar SHILUAN02-1 were obtained from Hebei Academy of Agricultural Sciences (Shijiazhuang, China). 

For measuring the antifungal activity of GO–Pyr against FG, a single floret injection was performed as described previously [34]. At the anthesis stage, point inoculation (10 μL mixture of spore suspension with 400 μg/mL GO, Pyr and GO–Pyr suspension, *v*:*v* = 1:1) was conducted on central spikelets of the selected spikes, which were then covered with small plastic bags for three days to maintain humidity for disease development. Some spikes were inoculated with MT in sterile water to be used as the negative control. In total, 90 spikes (30 for each subplot, three replicates) were evaluated. The spikelet disease was scored at 7 d after the inoculation. Quantitative infection symptoms, such as disease incidence (DI, the ratio of symptomatic spikelets to total spikelets) and disease index (DS), were visually determined based on a previously described 0–100% severity scale at 7 d after inoculation [35].

To measure the antifungal activity against *SS*, the Pyr and GO–Pyr solutions were diluted with MT solution to reach a final concentration of 200 μg/mL, which were then sprayed on 3-week-old oilseed rape plants. Thereafter, the SS mycelial agar/plugs (5 mm in diameter) were placed side down on oilseed rape leaves at 2 d after spraying and then incubated under light or dark conditions (25 °C and 85% humidity) [36]. The MT solution served as a control. The DI and DS were measured at 7 d after fungal challenge.

### 2.10. Statistical Analysis

SPSS version 11.5 (SPSS Inc., Chicago, IL, USA) was used for statistical analysis. Each treatment within a replicate was repeated for four times, with three replications. Data were presented as means ± SE and analyzed by using one-way ANOVA. Tukey’s HSD test was performed to test the significance. Statistically different from the control was considered at *p* < 0.05. The 50% effective concentration (EC_50_) was obtained by regressing of the percentage growth inhibition against the log-transformed fungicide concentration. The co-toxicity coefficient (CTC) was calculated with the equation [37]:

CTC = [EC_50_*A*/EC_50_(*A + B*)]/[(EC_50_*A*/EC_50_*A*) × *P_a_* + (EC_50_*A*/EC_50_*B*) × *P_b_*]. (5)

CTC significantly greater than 100 indicates synergistic interaction; that significantly lower than 100 represents antagonistic reaction; while that approximate to 100 indicates additive interaction.

## 3. Results and Discussion

### 3.1. Morphology of Formulated Graphene Oxide–Pyraclostrobin Nanocomposite Characterized by SEM

**Figure 1:**
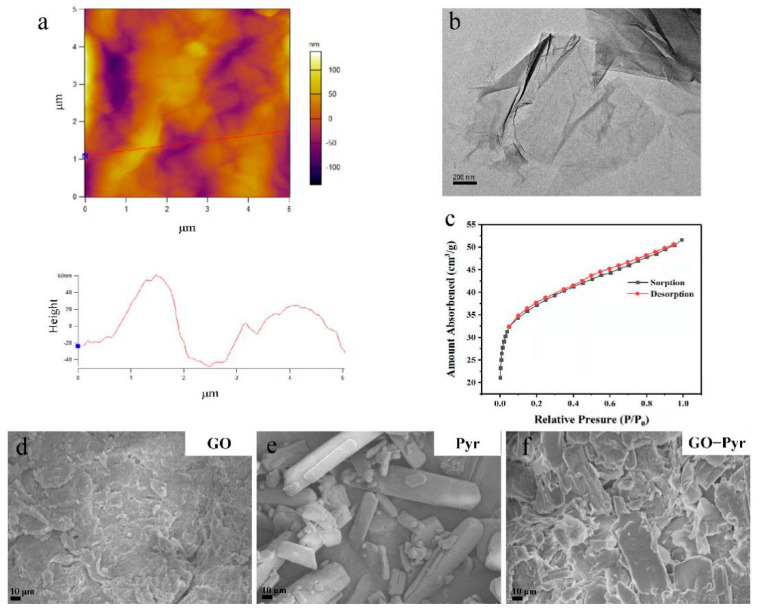
Morphological characterization of GO and formulated GO–Pyr nanocomposite. AFM images of GO (**a**), TEM images of GO (**b**), BET specific surface area of GO (**c**), SEM images of GO (**d**), Pyr (**e**), and GO–Pyr (**f**) at a mass ratio of 1:1.

The typical morphology of GO is displayed in Figure 1a and the images of GO were obtained using AEM. Single-layer GO was about 1–60 nm thick as observed from the representative AFM images. The 2D image of GO is shown in Figure 1b. The free-standing 2D GO sheets have flake-like shapes with high transparency and some wrinkles. The specific surface area of GO was calculated by the BET method by using nitrogen gas adsorption (Figure 1c). As a result, the value was as high as 137 m^2^/g, which is much higher than the previously reported values [38,39]. Such a large specific surface area of GO is beneficial to promote the adsorption of pesticides. Further, to compare the structural characteristics, the morphology of GO, Pyr and GO–Pyr nanocomposite with SEM was determined [40]. As shown in Figure 1d, GO exhibited a typical wrinkled morphology, while Pyr was crystal, characterized by a rectangular parallelepiped structure, smooth surface and different sizes (Figure 1e). Pyr loading led to the emergence of large amounts crystals with rectangular parallelepiped structure on the surface of GO (Figure 1f). The emergence of such crystals can be attributed to the adsorption of Pyr on GO sheets, which would be further verified by FT-IR later.

### 3.2. FT-IR Characterization of Graphene Oxide–Pyraclostrobin Nanocomposite

**Figure 2:**
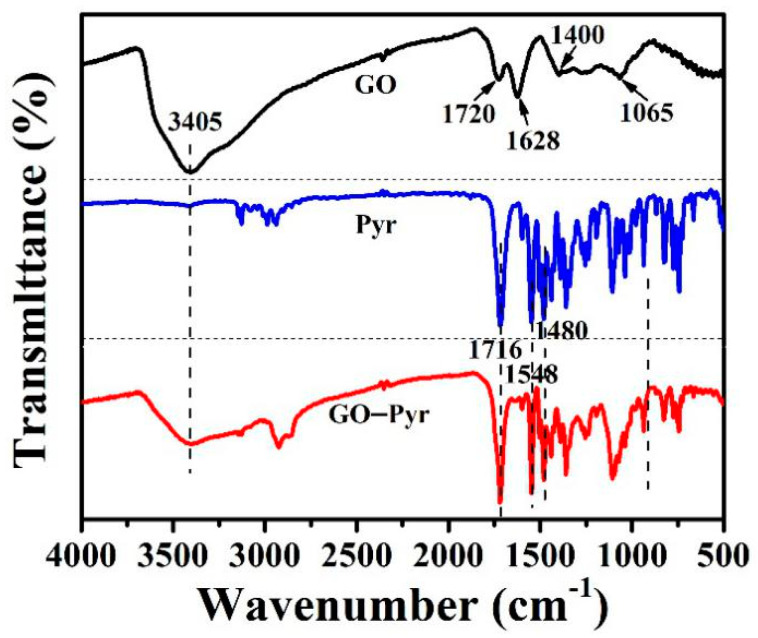
FT–IR spectra of GO, Pyr and GO–Pyr.

FT-IR spectra of GO, Pyr and GO–Pyr are shown in Figure 2. For GO, the peak at 3405 cm^−1^ belonged to the O-H stretching vibrations, the vibrations of graphite skeleton were located at 1628 cm^−1^, peaks at 1720 and 1065 cm^−1^ were contributed to COOH stretching vibrations and C-O stretching vibrations. In the FT-IR spectra for Pyr, stretching vibration of benzene skeleton was found at 1548 and 1480 cm^−1^, and the carbonyl stretching vibration of the ester group was observed at 1716 cm^−1^ [41,42]. Evidently, the spectra of GO–Pyr included all characteristic peaks of both GO and Pyr without a new peak, indicating physical loading of Pyr onto GO, which did not alter its chemical properties [43].

### 3.3. Thermal Stability Analysis of Graphene Oxide–Pyraclostrobin Nanocomposite

**Figure 3:**
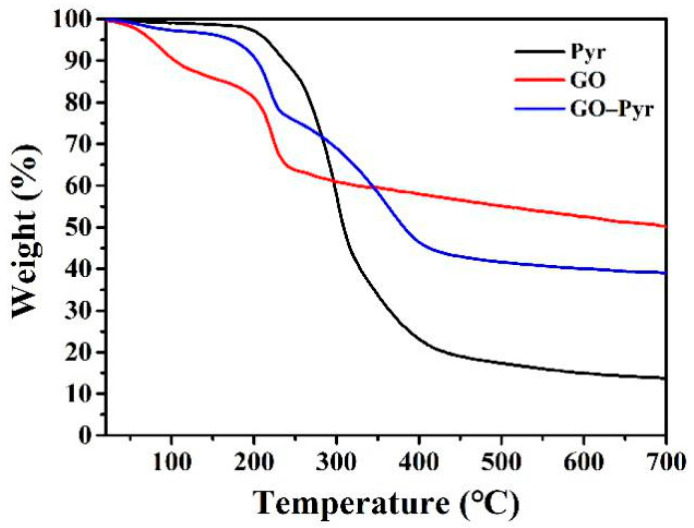
TGA curves of GO and GO–Pyr nanocomposite.

Thermal stability was analyzed by a comparison of the TGA curve between GO, Pyr and GO–Pyr nanocomposite (Figure 3). It can be observed that the weight loss of GO at 200 °C was 19.43%; the weight loss of Pyr at 200 °C was 2.87%; while that of GO–Pyr was only about 5%, suggesting that GO–Pyr nanocomposite had a higher thermal stability than GO while lower thermal stability than Pyr [44]. This phenomenon can be attributed to the weight loss of oxygen-containing functional groups in GO. According to the structure of Pyr, there was almost no loss of functional groups; while GO–Pyr nanocomposite had less weight loss than GO due to the occupation of part of the oxygen-containing functional groups by the H-bond interaction between Pyr and GO.

### 3.4. Loading Performance of Graphene Oxide for Pyraclostrobin

GO has various functional groups such as hydroxyl groups, which may be responsible for the heterogeneous adsorption of pesticides through the van der Waals force, hydrogen bonding and π–π interaction [45,46]. In the present work, the loading rate of Pyr at different Pyr:GO ratios was studied through centrifugation. The HPLC method was employed to determine the LC of Pyr on GO. Standard curves were obtained for Pyr at concentrations from 12.5 to 500 μg/mL, and the correlation coefficients were higher than 0.999 (Figure S2). The LC data are shown in Figure S3. At 50 μg/mL, the LC of Pyr on GO was 24.89%, and increased with increasing Pyr concentration. The LC tended to reach equilibrium at 250 μg/mL at the Pyr: GO ratio of 1:1, at which the LC was calculated to be 87.04%. The superior pesticide loading capacity of GO may be ascribed to the large surface and abundant functional groups on its surface, which can provide abundant adsorption sites for pesticide to realize efficient pesticide delivery and improve its bioavailability [23,47]. Moreover, a preliminary test to screen the optimal combined ratio of GO and Pyr revealed that the optimal ratio of GO and Pyr at 5:5 (equal to 1:1) can achieve the highest antifungal activity (Figure S1), which can be ascribed to the high LC of Pyr onto GO. A higher pesticide loading onto GO will bring about a higher antifungal activity of the nanocomposite. Therefore, the optimal GO: Pyr ratio was determined to be 1:1.

### 3.5. Release Behavior of Graphene Oxide–Pyraclostrobin Nanocomposite at Different Temperatures

**Figure 4:**
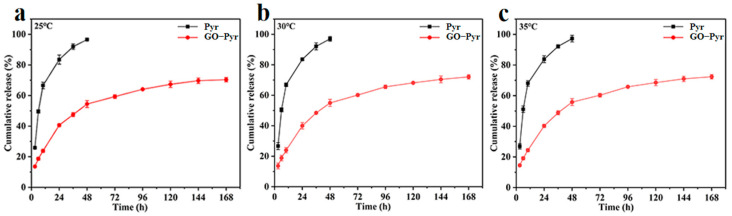
Release kinetics of Pyr and GO–Pyr nanocomposite at 25 °C (**a**), 30 °C (**b**) and 35 °C (**c**). Data are mean of cumulative release rate ± SE (*N* = 3).

Relative to traditional pesticide formulation, the nano-formulation of pesticide with smart vector can better control the release behavior and promote the bioavailability. To investigate the Pyr release behavior in vitro at different temperatures, Pyr and GO–Pyr nanocomposite were placed in a methanol–water mixture (1:1, *v*/*v*) at 25 °C, 30 °C and 35 °C for a week. The supernatant was collected at different time points to determine the amount of cumulative release by HPLC. The release behaviors of Pyr and GO–Pyr nanocomposite are presented in Figure 4 and Table S1. At the temperature of 25 °C, 30 °C and 35 °C, Pyr was released into the medium solution at a fast rate, with cumulative release rates of 96.58%, 97.00% and 97.26% in 48 h, respectively. In contrast, GO–Pyr displayed excellent sustained release of Pyr, which could still be observed even after 168 h. At the temperature of 25 °C, 30 °C and 35 °C, the release of Pyr from GO–Pyr was relatively fast in the first 48 h, with cumulative release rates of 54.47%, 54.99% and 55.81%, respectively; then, the release tended to be slow, with cumulative release of 70.38%, 72.09% and 72.35% after 168 h. The sustained release is consistent with the typical release pattern of controlled drug delivery systems. The first burst release and then slow release can maintain an effective concentration and high activities of the pesticide for a long time [48].

The Korsmeyer–Peppas model and Higuchi model, two classical kinetic models, were used to further investigate the sustained release of Pyr from the nanocomposite. The results are presented in Table 1. The Korsmeyer–Peppas model could provide better fitting of the data as indicated by its higher regression coefficients (*R*^2^) than the Higuchi model. The value of n was 0.32, 0.30 and 0.38, respectively, which were all lower than 0.43, indicating that the release of Pyr from the GO–Pyr nanocomposite follows the Fickian diffusion mechanism (*n* < 0.43), and the diffusion effect is the main factor for this release process [30].

### 3.6. Release Behavior of GO–Pyr Nanocomposite at Different pH

**Figure 5:**
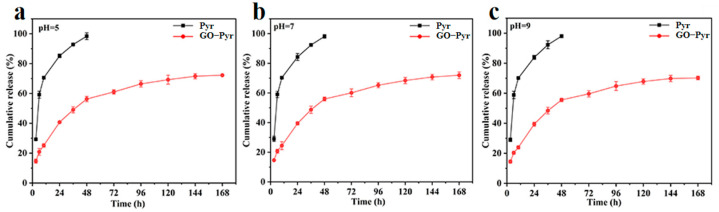
Release kinetics of Pyr and GO–Pyr nanocomposite at pH 5 (**a**), pH 7 (**b**) and pH 9 (**c**). Data are mean of cumulative release rate ± SE (*N* = 3).

The release behavior of Pyr from GO–Pyr nanocomposite in 30% ethanol aqueous solution under different pH (5.0, 7.0, and 9.0) at room temperature (25 ± 2 °C) was investigated. As shown in Figure 5 and Table S2, at 48 h, the cumulative release amount of Pyr from GO–Pyr nanocomposite was 56.33% (Figure 5a, pH 5.0), 55.94% (Figure 5b, pH 7.0), and 55.59% (Figure 5c, pH 9.0), respectively. At 168 h, the cumulative release amount was 72.24% (pH 5.0), 71.95% (pH 7.0), and 70.22% (pH 9.0), respectively. In contrast, at 48 h, the cumulative release amount of Pyr was 98.42% (pH 5.0), 98.17% (pH 7.0) and 98.03% (pH 9.0), respectively. The initial burst release and subsequent slow release of GO–Pyr nanocomposite may contribute to sustaining an effective concentration for a long time, which is conducive to the maintenance of high antifungal activity.

Similarly, the release kinetics of Pyr from GO–Pyr nanocomposite was analyzed using the Higuchi and Korsmeyer–Peppas model. The values of related parameters and regression coefficients (*R*^2^) are presented in Table 2. The Korsmeyer–Peppas model could achieve better fitting of the data with higher *R*^2^ than the Higuchi model. Moreover, the values of relevant indices (*n* = 0.3271, 0.307 and 0.3873) were all lower than 0.43 at pH 5.0, 7.0 and 9.0, indicating that Pyr release from the nanocomposite under these conditions can be attributed to the Fickian diffusion mechanism. In other words, Pyr is released mainly through the diffusion effect [30].

### 3.7. Storage Stability of Graphene Oxide–Pyraclostrobin Nanocomposite

The storage stability is a main indicator to evaluate the quality of pesticide formulation, which is critical for the effective application after storage and during the spraying process [22,49]. Hence, the stability of the GO–Pyr nanocomposite was assessed by monitoring the contents of effective Pyr components during two-year storage as well as under low- or high-temperature storage.

**Figure 6:**
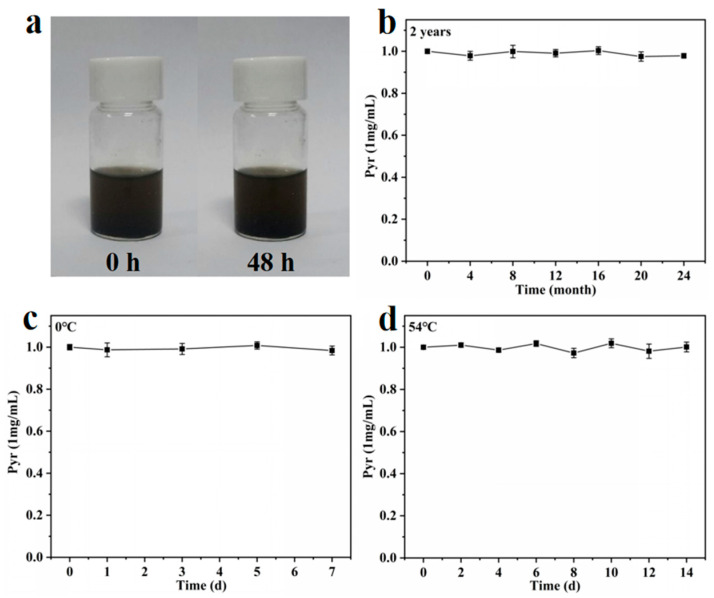
Storage stability of GO–Pyr nanocomposite. (**a**) Photos of GO–Pyr at 0 and 48 h, (**b**) stability of long-term storage, (**c**) storage stability under low temperature, and (**d**) storage stability under high temperature.

The colloidal stability of GO–Pyr nanocomposite was studied by standing at room temperature for 0 and 48 h. As shown in Figure 6a, the as-prepared GO–Pyr nanocomposite presented a clear and uniform black solution without flocculation or precipitation. In addition, the content of Pyr showed nearly negligible changes during the two-year storage (Figure 6b). The images of GO–Pyr nanocomposite after low- and high-temperature storage are shown in Figure S4. It can be seen that all the GO–Pyr nanocomposite stayed stable with no precipitation or stratification during storage (Figure 6c,d), which confirms the stability of the GO–Pyr nanocomposite. Moreover, after two years of storage, despite of slight precipitation, the nanocomposite could still be evenly dispersed after shaking, which meets the requirements of real production and application (Figure S5).

### 3.8. Bioactivity Assay of Graphene Oxide–Pyraclostrobin Nanocomposite In Vitro

**Figure 7:**
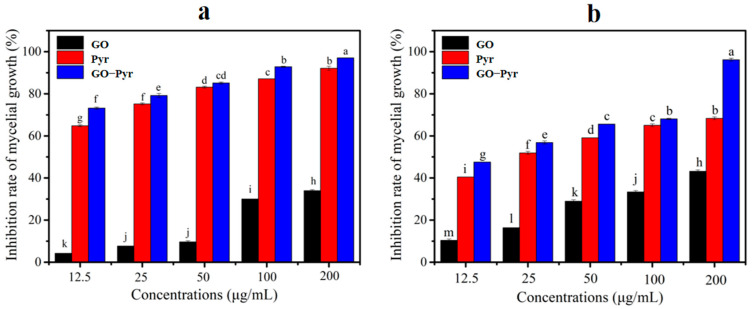
Synergistic effects of GO, Pyr and GO–Pyr nanocomposite on the mycelial growth rate of FG (**a**) and SS (**b**). Different lower case letters indicate significant differences between treatments (*p* < 0.05).

Figure 7 presents the inhibitory effects of GO, Pyr and GO–Pyr nanocomposite on the mycelial growth of FG and SS at different concentrations. Figure 7a shows that at 12.5–200 μg/mL, GO could have 4.22–34% inhibition rates on the mycelial growth of FG; while Pyr could achieve inhibition rates of 64.76–92.06%. Notably, the efficacy of controlling the mycelial growth of FG of GO–Pyr nanocomposite was much higher than that of GO and Pyr alone. It could inhibit the mycelial growth by 73.20% at 12.5 μg/mL, and the inhibition rate could even reach 97.03% at the 200 μg/mL, confirming that the nanocomposite has a much stronger antifungal effect than Pyr and GO alone. Similar results could be observed in Figure 7b. The GO–Pyr nanocomposite also showed higher antifungal activity than GO and Pyr against the mycelial growth of SS. In addition, all GO, Pyr and GO–Pyr nanocomposites had certain inhibitory effects on the mycelial growth in a dose-dependent manner.

Table 3 shows the EC_50_ values of Pyr and GO–Pyr nanocomposite against the mycelial growth of FG and SS obtained from a probit analysis with a 95% confidence limit. The results indicated that the combination of GO and Pyr (1:1) resulted in a synergistic antifungal effect on both the mycelial growth of FG and SS, with the CTC value exceeding 100.

### 3.9. Control Efficacy of Graphene Oxide–Pyraclostrobin Nanocomposite on FG and SS in the Greenhouse

As shown in Table 4, the GO–Pyr nanocomposite can significantly decrease the DI and DS of FG and SS in the greenhouse. The GO–Pyr nanocomposite could significantly reduce the DI relative to the MT in sterile water (CK). The GO–Pyr nanocomposite showed significant control efficacy of 71.35% and 62.32% for FG and SS (*p* < 0.05), respectively. Particularly, the GO–Pyr nanocomposite could remarkably decrease the infection of FG and SS, demonstrating a higher antifungal activity than single Pyr. Therefore, GO may be a promising synergist to be used for Pyr in controlling plant fungal pathogens.

### 3.10. Adhesion Ability of Graphene Oxide–Pyraclostrobin Nanocomposite

**Figure 8:**
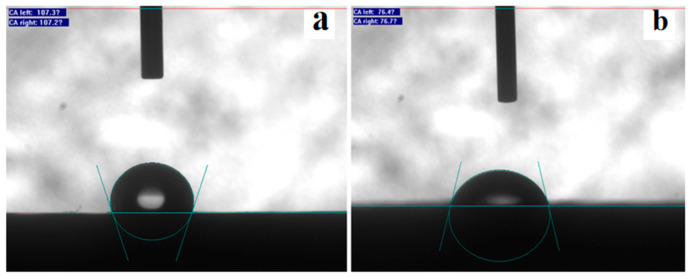
Contact angles of (**a**) water and (**b**) GO–Pyr nanocomposite on the surface of paraffin film.

Wettability and retention on leaf surface after spraying are important factors affecting the utilization rate of pesticide [49]. Hence, the wetting characteristics and adhesion ability of GO–Pyr nanocomposite by measuring the contact angle were evaluated. The contact angles of water and GO–Pyr nanocomposite on the surface of paraffin film were about 107° and 74°, respectively (Figure 8). The low contact angle demonstrated that the GO–Pyr nanocomposite has strong adhesion ability and spreadability on paraffin film surface, which can facilitate the adsorption and deposition of the pesticide.

## 4. Conclusions

This study develops a GO-based nanocarrier for pesticide delivery and pest control. The prepared GO–Pyr nanocomposite showed high solubility and stability in aqueous solutions, which is conducive to the dispersion and utilization of the pesticide. In addition, GO–Pyr exhibited a good release behavior, indicating a sustained release of Pyr from the nanocomposite. Due to the above improvement of properties, the GO–Pyr nanocomposite has satisfactory antifungal activity against FG and SS. Considering the simple preparation, high antifungal activity and avoidance of toxic organic solvents and additives, the pesticide delivery system formulated with GO as the nanocarrier may hold a great promise in future plant protection and sustainable agriculture.

## Figures and Tables

**Table 1:** Kinetic parameters of Pyr release from GO–Pyr nanocomposite at 25 °C, 30 °C and 35 °C.

Conditions		Higuchi Model		Korsmeyer-Peppas Model		
		*K* (d^−0.5^)	*R* ^2^	*K* (d^−n^)	*n*	*R* ^2^
25 °C	Pyr	13.9718	0.9947	16.1247	0.4625	0.9956
	GO–Pyr	7.6128	0.5861	14.2589	0.3271	0.9561
30 °C	Pyr	16.6662	0.7095	29.7182	0.3195	0.9157
	GO–Pyr	6.7779	0.6776	15.7871	0.3073	0.9645
35 °C	Pyr	15.7556	0.8613	25.2932	0.3614	0.9242
	GO–Pyr	6.6689	0.9384	10.9860	0.3873	0.9772

**Table 2:** Kinetic parameters of Pyr release from GO–Pyr nanocomposite at pH 5.0, 6.0 and 7.0.

Conditions		Higuchi Model		Korsmeyer-Peppas Model		
		*K* (d^−0.5^)	*R* ^2^	*K* (d^−n^)	*n*	*R* ^2^
pH 5.0	Pyr	16.1224	0.9470	19.9661	0.4332	0.9527
	GO–Pyr	5.5989	0.8737	14.6924	0.3110	0.9833
pH 7.0	Pyr	18.6739	0.8523	39.8791	0.2385	0.8612
	GO–Pyr	7.0909	0.9257	9.6202	0.4154	0.9792
pH 9.0	Pyr	20.3456	0.8946	38.3565	0.2553	0.9741
	GO–Pyr	6.3901	0.8734	11.8248	0.3619	0.9566

**Table 3:** Synergistic effects of GO–Pyr nanocomposite on the mycelial growth of FG and SS.

Fungi	Treatment	Slop ± SE ^a^	EC_50_(μg/mL)(95% CL) ^b^	CTC ^c^
*FG*	GO	1.17 ± 0.19 0.83 ± 0.04	415.19(200.41 ~ 860.14)	- -
	Pyr		4.07(2.37 ~ 6.96)	
	GO–Pyr	1.08 ± 0.11	4.03(3.11 ~ 5.24)	196.65
*SS*	GO	0.90 ± 0.08 0.59 ± 0.06	281.57(195.44 ~ 405.66)	- -
	Pyr		24.82(18.80 ~ 32.78)	
	GO–Pyr	1.32 ± 0.41	19.10(8.18 ~ 44.58)	238.92

^a^ Slope of the probit mortality line. ^b^ EC_50_ values and data in brackets are 95% confidence limits (CL). ^c^ According to the CTF formula, CTC significantly greater than 100 indicates synergistic interaction; that significantly lower than 100 represents antagonistic interaction; and that approximate to 100 indicates additive interaction.

**Table 4:** Control efficacy of GO, Pyr and GO–Pyr nanocomposite on FG and SS under greenhouse conditions (25 °C and 85% humidity).

Fungi	Treatment (200 μg/mL)	Disease Incidence (%) (7d)	Disease Severity (%) (7d)	Control Efficacy (%)
*FG*	CK	82.67 ± 0.71a	33.62 ± 1.21a	-
	GO	48.67 ± 2.12b	27.44 ± 2.79a	15.66c
	Pyr	30.33 ± 0.71c	17.98 ± 2.02b	43.71b
	GO–Pyr	24.67 ± 2.83c	8.66 ± 0.57c	71.35a
*SS*	CK	89.00 ± 1.41a	27.92 ± 1.47a	-
	GO	74.33 ± 2.12ab	19.17 ± 1.05b	27.77c
	Pyr	54.67 ± 0.71bc	16.43 ± 1.76b	37.58b
	GO–Pyr	36.67 ± 9.89c	9.52 ± 0.18c	62.32a

Different lower case letters indicate significant differences between treatments (*p* < 0.05).

## Data Availability

Data presented in this article are available at request from the corresponding author.

## Supplementary Materials

The following supporting information can be downloaded at: https://www.mdpi.com/article/10.3390/nano12071112/s1, Table S1: Release kinetics of Pyr and GO-Pyr nanocomposite at 25 °C, 30 °C and 35 °C. Data are mean of cumulative release rates ± stand error (SE). Error bars represent the SE (*N* = 3); Table S2: Release kinetics of Pyr and GO-Pyr nanocomposite at pH 5, pH 7 and pH 9. Data are mean of cumulative release rates ± SE. Error bars represent the SE (*N* = 3); Figure S1: Inhibitory activities of single fungicides or in combination with GO at different mass ratios against mycelial growth of F. graminearum. Data are mean ± stand error (SE). Error bars represent the SE (*N* = 3). Different lower case letters indicate significant differences between treatments (*p* < 0.05); Figure S2: HPLC standard curves of Pyr; Figure S3: Loading capacity of Pyr on GO at different concentration ratio; Figure S4: Images of GO-Pyr under storage of 0 °C and 54 °C; Figure S5: Images of GO-Pyr in 2 years of storage.
