# Supplementary material for: Nanopesticide Formulation from Pyraclostrobin and Graphene Oxide as a Nanocarrier and Application in Controlling Plant Fungal Pathogens
Source: Nanomaterials (Basel). 2022 Mar 28;12(7):1112. doi: 10.3390/nano12071112 (PMC9000337; doi:10.3390/nano12071112)
Supplement: Supplementary file 1 [file nanomaterials-12-01112-s001.zip › nanomaterials-1621733-supplementary.pdf]

nanomaterials

Supplementary Materials

Nanopesticide Formulation from Pyraclostrobin and Graphene Oxide as a Nanocarrier and Application in Controlling Plant Fungal Pathogens

Fei Peng [1,2,†], Xiuping Wang [2,3,†], Wenjing Zhang [1], Xuejuan Shi [3], Caihong Cheng [1,2], Wenlong Hou [1,2], Xiaohu Lin [2], Xiaolu Xiao [4] and Jun Li [4,*]

[1] Hebei Key Laboratory of Active Components and Functions in Natural Products, Hebei Normal University of Science and Technology, Qinhuangdao 066004, China; flyer5212528@163.com (F.P.); zwj19990123@126.com (W.Z.); cch20059@126.com (C.C.); wenlonghou@126.com (W.H.)
[2] Analysis and Testing Center, Hebei Normal University of Science and Technology, Qinhuangdao 066000, China; wangxiuping0721@163.com (X.W.); xiaohulin2008@163.com (X.L.)
[3] College of Agronomy and Biotechnology, Hebei Normal University of Science and Technology, Qinhuangdao 066000, China; xuejuanshi@163.com
[4] Key Laboratory of Biology and Genetic Improvement of Oil Crops of Ministry of Agriculture and Rural Affairs, Oil Crops Research Institute of the Chinese Academy of Agricultural Sciences, Wuhan 430062, China; xiaoxiaoluxt@163.com (X.X.)
* Correspondence: lijun02@caas.cn; Tel. +86-027-867-11182
† These authors contributed equally to this work.

Citation: Peng, F.; Wang, X.; Zhang, W.; Shi, X.; Cheng, C.; Hou, W.; Lin, X.; Xiao, X.; Li, J. Nanopesticide Formulation from Pyraclostrobin and Graphene Oxide as a Nanocarrier and Application in Controlling Plant Fungal Pathogen. *Nanomaterials* **2022**, *12*, 1112. https://doi.org/10.3390/nano12071112

Academic Editor: Marcelo Antunes

Received: 17 February 2022
Accepted: 22 March 2022
Published: 28 March 2022

Publisher's Note: MDPI stays neutral with regard to jurisdictional claims in published maps and institutional affiliations.

Copyright: © 2022 by the authors. Submitted for possible open access publication under the terms and conditions of the Creative Commons Attribution (CC BY) license (https://creativecommons.org/licenses/by/4.0/).

Table S1. Release kinetics of Pyr and GO-Pyr nanocomposite at 25 °C, 30 °C and 35 °C . Data are mean of cumulative release rates ± stand error (SE). Error bars represent the SE (N = 3).

Temperature (°C)		the cumulative release rate at t (h)										
		3	6	10	24	36	48	72	96	120	144	168
25°C	pyr	25.91±0.23	49.65±1.11	66.60±2.10	83.51±2.98	91.99±1.80	96.58±0.22	—	—	—	—	—
	GO-pyr	13.63±2.09	18.69±1.19	23.87±2.21	40.64±0.35	47.55±1.24	54.47± 2.79	59.33±1.02	64.13±2.34	67.37±1.43	69.71±2.57	70.38±1.73
30°C	pyr	26.69±2.30	50.50±1.34	66.91±1.20	83.59±0.75	92.10±2.27	97.01±1.58	—	—	—	—	—
	GO-pyr	13.68±1.89	18.86±1.86	23.96± 1.78	40.00±2.17	48.45±0.38	54.99±2.31	60.17±0.86	65.54±1.22	68.18±0.45	70.44±2.23	72.09±1.45
35°C	pyr	26.95±1.78	51.17±2.16	68.03±1.78	83.82±2.24	92.19±1.10	97.26±1.96	—	—	—	—	—
	GO-pyr	14.52±0.84	19.14±0.91	24.43±1.04	40.27±0.98	48.86±1.26	55.81±2.30	60.31±1.21	65.81±0.65	68.53±2.10	70.99±1.78	72.35±1.45

Table S2. Release kinetics of Pyr and GO-Pyr nanocomposite at pH 5, pH 7 and pH 9 . Data are mean of cumulative release rates ± stand error (SE). Error bars represent the SE (N = 3).

pH		the cumulative release rate at t (h)										
		3	6	10	24	36	48	72	96	120	144	168
5	pyr	29.30± 0.40	59.24± 2.18	70.59 ±0.85	85.24±1.27	92.82±0.54	98.42±2.12	—	—	—	—	—
	GO-pyr	14.70±1.35	20.92±2.33	25.20±1.18	40.76±0.69	49.09±2.12	56.33±1.77	61.08±1.50	66.45±2.15	69.25±2.97	71.59±1.74	72.24±0.43
7	pyr	29.06±1.73	59.03±3.65	70.32±0.36	84.29±2.30	92.38±0.87	98.17±1.20	—	—	—	—	—
	GO-pyr	14.67±0.33	20.75±1.36	25.48±2.68	39.51±1.00	48.71±2.36	55.94±1.28	60.12±2.64	65.25±1.75	68.40±2.10	70.77±1.87	71.95±2.12
9	pyr	29.10±1.23	58.88±2.48	70.14±0.30	83.93±1.32	92.30±2.62	98.03±1.09	—	—	—	—	—
	GO-pyr	14.50±1.02	20.37±1.07	23.97±1.04	39.45±1.32	48.43±2.33	55.59±1.15	59.65±2.07	64.82±2.95	67.86±1.73	69.81±2.11	70.22±1.22

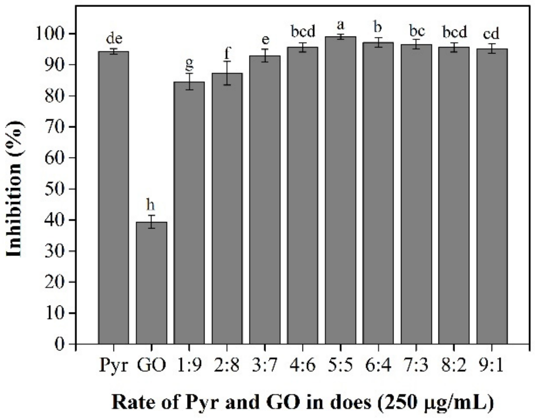

Figure S1. Inhibitory activities of single fungicides or in combination with GO at different mass ratios against mycelial growth of *F. graminearum*. Data are mean ± stand error (SE). Error bars represent the SE (N = 3). Different lower case letters indicate significant differences between treatments (P < 0.05).

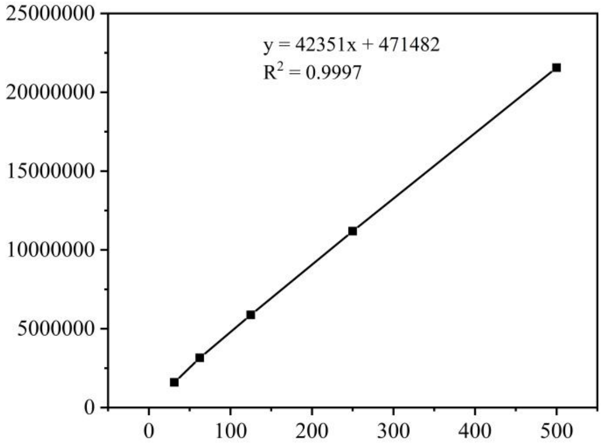

Figure S2. HPLC standard curves of Pyr.

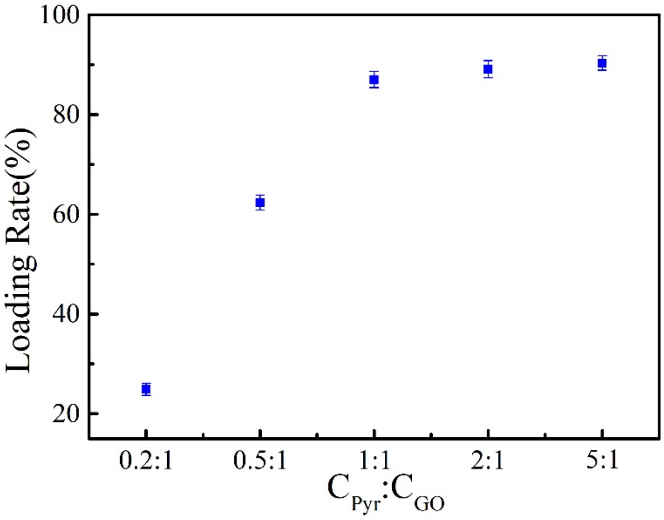

Figure S3. Loading capacity of Pyr on GO at different concentration ratio.

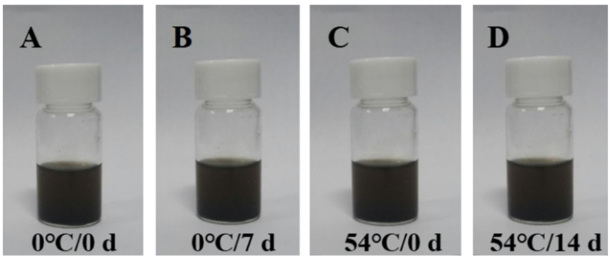

Figure S4. *Images of GO-Pyr under storage of 0 °C and 54 °C.*

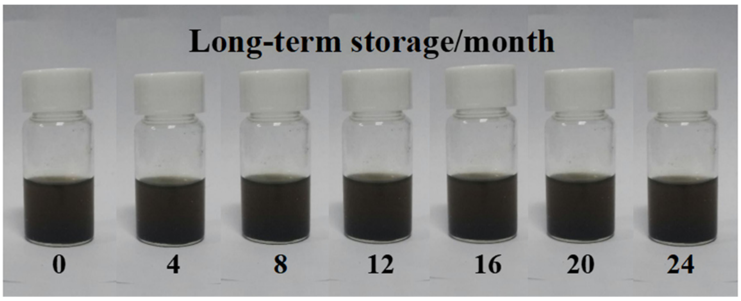

Figure S5. *Images of GO-Pyr in 2 years of storage.*
